# Evaluation of the DendrisKIT^®^DP for the Diagnosis of Superficial Fungal Infections

**DOI:** 10.3390/jof11040269

**Published:** 2025-04-01

**Authors:** Pauline Tirard-Collet, François Durupt, Marion Hérault, Charline Miossec, Jean-Philippe Lemoine, Martine Wallon, Damien Dupont, Florence Persat, Jean Menotti

**Affiliations:** 1 Service de Parasitologie et de Mycologie Médicale, Institut des Agents Infectieux, Hôpital de la Croix Rousse, Hospices Civils de Lyon, Université Claude Bernard—Lyon 1, 69004 Lyon, France; pauline.tirard-collet@chu-lyon.fr (P.T.-C.);; 2 Service de Dermatologie, Hôpital de la Croix Rousse, Hospices Civils de Lyon, 69004 Lyon, France

**Keywords:** superficial fungal infection, dermatophytosis, PCR kit, molecular diagnosis, machine learning

## Abstract

Conventional diagnosis of fungal infections of the skin, nail, and hair requires both expertise in mycology and prolonged cultures. We evaluated a new molecular tool based on an innovative technology, the DendrisKIT^®^DP, combining a pan-fungal PCR, a DNA chip and a decision algorithm using machine learning, for the diagnosis of superficial fungal infections directly from clinical samples. It enables the simultaneous detection of *Candida albicans* and twelve dermatophytes, providing faster results than conventional techniques. Among 85 clinical samples (50 skin scrapings, 29 nail specimens, and 6 hair specimens) routinely tested by microscopic examination and cultures that were retrospectively tested by the DendrisKIT^®^DP, we found a sensitivity of 83.9% and a specificity of 88.9%. This performance appeared satisfactory compared to microscopy and culture, and results were achieved much faster than with cultures, saving time for patient management. Moreover, thanks to the continuous improvement in the identification algorithm due to enriching the database, its performance is likely to be further enhanced.

## 1. Introduction

Mainly caused by dermatophytes, superficial fungal infections (SFI) affect the skin, hair, and nails, leading to frequent dermatological consultations due to the functional and esthetic impact on millions of patients worldwide [1,2]. Reference diagnostic methods—microscopic examination (ME) and conventional culture—have a low sensitivity. Moreover, cultures can take up to four weeks before yielding a result, and their performances can be impaired by sub-optimal sampling as distal nail sampling or concomitant anti-fungal intake. Over the past two decades, various PCR techniques have been developed to overcome these limitations, but they often do not allow species identification without sequence analysis or allow a limited number of species detection and identification [3,4,5,6,7,8]. Herein, we retrospectively evaluated the DendrisKIT^®^DP, a new syndromic molecular test based on an innovative technology combining a pan-fungal PCR, a DNA microchip, and a machine-learning-based decision algorithm. It enables the simultaneous detection of *Candida albicans*, *Epidermophyton floccosum*, *Microsporum* spp., *Nannizzia gypsea*, *Trichophyton benhamiae*, *Trichophyton mentagrophytes/Trichophyton interdigitale*, *Trichophyton rubrum*, *Trichophyton soudanense*, *Trichophyton tonsurans*, *Trichophyton verrucosum, Trichophyton violaceum,* and other *Trichophyton* spp. [9].

## 2. Materials and Methods

A total of 85 clinical samples (50 skin scrapings, 29 nail specimens, and 6 hair specimens), collected as part of the SFI diagnostic activity of the Hospices Civils de Lyon mycology laboratory, were selected during a six-month period from 31 July 2023 to 26 January 2024 to be retrospectively tested with the DendrisKIT^®^DP. The routine laboratory workflow was based on the ME of mounted samples with 10% potassium hydroxide and calcofluor white (Becton Dickinson, Franklin Lakes, NJ, USA), and on sample cultures in Sabouraud agar with chloramphenicol ± cycloheximide (bioMérieux, Marcy l’Etoile, France) incubated at 25 °C for three weeks and examined twice a week. Yeasts were identified at the species level by MALDI-TOF with a Vitek MS (bioMérieux) using the Vitek MS and MSI-2 [10] databases. Filamentous fungi were identified on the basis of macroscopic and microscopic morphological features and, if necessary, by MALDI-TOF after formic acid/acetonitrile protein extraction and/or by *ITS 1–2* sequencing with Sanger technology (Microsynth, Vaulx-en-Velin, France).

After overnight incubation of samples in Dendris pre-treatment solution, DNA was manually extracted using NucleoSpin^®^ gDNA clean-up columns (Macherey-Nagel, Hœrdt, France). Real-time PCR was performed according to the manufacturer’s instructions on a CFX96 thermocycler (BioRad, Marnes-la-Coquette, France) using the seven fungus-specific primer pairs included in the kit to amplify the complete *ITS* sequence. Human *18S* gene was amplified as an extraction and inhibition control (IC). Amplicons were purified and hybridized onto the chip by the automated DendriSTATION^®^ (Dendris, Labège, France), according to the manufacturer’s instructions. On-chip hybridization profiles were read using the DendriSCAN^®^ reader and analyzed with the DendriSOFT^®^ software version 1.0.0. by machine-learning algorithms using a hierarchy of multilabel classifier combined with a random forest multilabel version [11,12]. For negative samples with IC Ct > 37, PCR and hybridization were repeated with half- and tenfold-diluted DNA to remove potential polymerase inhibitors; in the case of IC Ct still superior to 37, PCR was considered invalid.

In case of unexpected kit identification, i.e., kit positive result with negative culture or species identification discrepancy, a panfungal PCR was performed using MITS 2a/2b primers [13] followed by Sanger sequencing (Microsynth, France), using a 98% similarity threshold for fungal identification.

To assess sensitivity and specificity, reference results were defined as panfungal PCR followed by DNA sequencing identification when performed and successful; or as culture identification otherwise, considering only the fungal species targeted by the kit panel to assess the performance based solely on the intrinsic characteristics of the test. Therefore, the absence of detection by the DendrisKIT^®^DP of fungi not included in the kit panel was considered a true negative result. Samples with invalid PCR results were excluded from the analysis. Confidence intervals were estimated using the Wilson score interval.

## 3. Results

By culture, the samples were positive for dermatophytes and/or *C. albicans* (*n* = 57), non-dermatophytes/non-*C. albicans* fungi (*n* = 8), or sterile (*n* = 20; Table 1). The DendrisKIT^®^DP identified dermatophytes and/or *C. albicans* in 54 (63.5%) samples. A negative result was obtained in 26 (30.6%) samples and PCR was invalid in five (5.9%). Among the 57 samples associated with dermatophytes and/or *C. albicans* culture, the DendrisKIT^®^DP was positive for 44 (77.2%) samples—including three samples with a species identification different from that identified by culture—but negative for ten (17.5%) samples, all being skin scrapings and nine having a positive ME. PCR was invalid for three samples.

Among the eight samples associated with non-dermatophytes/non-*C. albicans* culture, the DendrisKIT^®^DP was negative for five (62.5%) samples but positive for two (25.0%). PCR was invalid for one sample. Among the 20 samples with a sterile culture, 11 (55.0%) had a negative DendrisKIT^®^DP result, but 8 (40.0%) had an unexpected positive DendrisKIT^®^DP result, including 2 with a negative ME; PCR was invalid for 1 sample (Table 1 and Supplementary Table S1).

**Figure 1:**
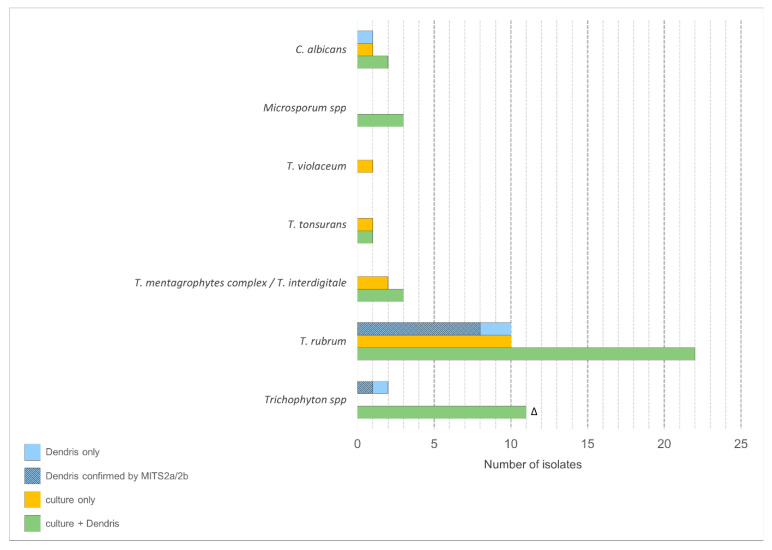
Isolate counts for each identified species according to the method. Δ Identified as *Trichophyton* spp by DendrisKIT^®^DP and as *T. rubrum* (*n* = 6), *T.mentagrophytes complex* (*n* = 3), *T. interdigitale* (*n* = 2) by culture.

MITS 2a/2b sequencing confirmed the DendrisKIT^®^DP identification in 9 of the 13 samples tested and was inconclusive for the remaining 4 (Figure 1).

Overall, when considering culture and MITS 2a/2b sequencing results, the DendrisKIT^®^DP results were concordant for 65 (76.5%) samples, with a sensitivity of 83.9% (95% confidence interval (CI); 72.8–91.0) and a specificity of 88.9% (95%CI; 67.2–96.9).

## 4. Discussion

Other studies in the literature have evaluated the performances of in-house PCRs and PCR kits for the detection of dermatophytes, although some studies have focused on the diagnosis of onychomycosis only. For example, Kabtani et al. [6] evaluated the EurobioPlex Dermatophyte Real-Time PCR, detecting only six dermatophytes, and found a low sensitivity of 65.4%. The in-house real-time PCR evaluated by Paugam et al. [4] demonstrated a sensitivity of 79%, similar to our findings, while the one evaluated by Hafirassou et al. [5], specifically on nail samples, showed a higher sensitivity of 90%. However, both studies required an additional sequencing step. Recently, Evrard et al. [8] reported a similar sensitivity of 78.2–82.7% (depending on pathogen classification) using the DermaGenius^®^2.0 PCR. The Novaplex dermatophyte assay, evaluated by Harel et al. [7], showed a promising sensitivity of 92.9% but lacks the advantages of microarray technologies like the DendrisKIT^®^DP, such as the easier implementation of new species detection through its identification algorithm. Finally, the EUROArray dermatomycosis kit, also based on microarray technology, showed slightly lower sensitivity and specificity in the study by Trave et al. [14]. Our evaluation of the DendrisKIT^®^DP, in comparison with other available technologies, therefore highlights its satisfactory performance for the diagnosis of SFI directly from patient samples. Among the 85 samples, it failed to detect 10 dermatophytes compared to cultures but allowed the additional identification of 11 dermatophytes not detected by cultures, 9 of which were confirmed by MITS 2a/2b sequencing. These discordant results may be partly explained by a heterogeneous fungal distribution within the sample and by the need to collect alive fungi to obtain a culture, contrary to molecular methods.

The main indication for this kit should be clinical suspicion of dermatophytosis, as these infections are difficult to diagnose and treat, whereas mucocutaneous candidiasis is usually easily diagnosed by conventional methods and generally responds well to anti-fungal treatment. Indeed, the major advantage of the DendrisKIT^®^DP is the time saved to reach dermatophytosis diagnosis, although this could not be assessed in our retrospective study. However, the DendrisKIT^®^DP should provide faster results for dermatophyte detection than cultures, since its theoretical turnaround time is below 48 h [9]. Therefore, the DendrisKIT^®^DP may enable to improve the management of dermatophytosis, especially as it is compatible with recent or concomitant anti-fungal medication, thus avoiding a therapeutic window before sampling and saving time for patient management. However, it should not be forgotten that this kit panel does not allow the detection of pseudo-dermatophytes, such as *Scytalidium hyalinum* and *Neoscytalidium dimidiatum*, and non-dermatophyte molds, such as *Fusarium* sp., that may also be involved in onychomycosis or skin infections. Therefore, we believe that a new version of the microarray incorporating the relevant non-dermatophyte molds should be considered to enhance diagnostic performance, with the corresponding profiles easily added to the database by the manufacturer.

## 5. Conclusions

Incorporating this technology in a new diagnostic strategy seems to be promising to save time for patient management. Our findings suggest that the diagnosis of superficial fungal infection should not rely on this test alone and that conventional methods remain important to ensure optimal sensitivity—especially for fungi not included in the kit panel—and to allow anti-fungal sensitivity testing if necessary. A diagnostic algorithm with a first step using DendrisKIT^®^DP for rapid results and species identification, followed by conventional methods if the DendrisKIT^®^DP result is negative, could be considered and evaluated by a cost-effectiveness study. It is noteworthy that since this new technology includes the continuous improvement of the identification algorithm through database enrichment, its performance is likely to be further improved.

## Figures and Tables

**Table 1:** Results obtained with the DendrisKIT^®^DP according to culture category and sample type.

Culture Category		Total (*n*)	Skin (*n*)	Nail (*n*)	Hair (*n*)
Samples with dermatophytes and/or *C. albicans* culture		57	40	11	6
	Kit positive	44 *	28 *	10	6
	ME pos	38 *	25 *	9	4
	ME neg	4	1	1	2
	ME:IQ	2	2	0	0
	Kit negative	10	10	0	0
	ME pos	9	9		
	ME neg	1	1		
	ME:IQ	0	0		
	Invalid PCR	3	2	1	0
	ME pos	2	1	1	
	ME neg	0	0	0	
	ME:IQ	1	1	0	
Samples with non-dermatophytes/non-*C. albicans* culture		8	5	3	0
	Kit negative	5	2	3	0
	ME pos	3	0	3	
	ME neg	2	2	0	
	ME:IQ	0	0	0	
	Kit positive	2	2	0	0
	ME pos	1	1		
	ME neg	1	1		
	ME:IQ	0	0		
	Invalid PCR	1	1	0	0
	ME pos	0	0		
	ME neg	1	1		
	ME:IQ	0	0		
Samples with sterile culture		20	7	13	0
	Kit negative	11	5	6	0
	ME pos	4	2	2	
	ME neg	7	3	4	
	ME:IQ	0	0	0	
	Kit positive	8	2	6	0
	ME pos	6	1	5	
	ME neg	2	1	1	
	ME:IQ	0	0	0	
	Invalid PCR	1	0	1	0
	ME pos	0		0	
	ME neg	1		1	
	ME:IQ	0		0	

IQ: insufficient quantity; ME: microscopic examination; neg: negative; pos: positive. * For one skin sample with *Candida albicans* in culture, the DendrisKIT^®^DP unexpectedly identified *Trichophyton rubrum*. The kit identification was confirmed by the presence of hyphae on direct microscopic examination and by the identification of *T. rubrum* by DNA sequencing after panfungal PCR with MITS2a/2b primers.

## Data Availability

The original contributions presented in this study are included in the article/Supplementary Material. Further inquiries can be directed to the corresponding author.

## Supplementary Materials

The following supporting information can be downloaded at: https://www.mdpi.com/article/10.3390/jof11040269/s1, Table S1: Characterization of the 85 clinical samples retrospectively tested with the DendrisKIT^®^DP.
