# Supplementary material for: Evaluation of the DendrisKIT®DP for the Diagnosis of Superficial Fungal Infections
Source: J Fungi (Basel). 2025 Apr 1;11(4):269. doi: 10.3390/jof11040269 (PMC12028271; doi:10.3390/jof11040269)
Supplement: Supplementary file 1 [file jof-11-00269-s001.zip › jof-3531856-supplementary.pdf]

Table S1. Characterization of the 85 clinical samples retrospectively tested with the DendrisKIT®DP

Sample number	Sample type	Culture	DendrisKIT®DP	MITS2a/2b sequencing	DME
1	Skin	*Candida albicans*	*Candida albicans*		pseudohyphae
2	Skin	*Candida albicans*	*Candida albicans*		pseudohyphae
3	Skin	*Candida albicans*	*Trichophyton rubrum*	*Trichophyton rubrum*	hyphae
4	Hair	*Microsporum canis*	*Microsporum* sp.		hyphae
5	Hair	*Microsporum canis*	*Microsporum* sp.		neg
6	Hair	*Microsporum canis*	*Microsporum* sp.		hyphae
7	Skin	*Trichophyton indotineae*	*Trichophyton mentagrophytes* complex/*Trichophyton interdigitale*		hyphae
8	Skin	*Trichophyton indotineae*	*Trichophyton mentagrophytes* complex/*Trichophyton interdigitale*		hyphae
9	Skin	*Trichophyton indotineae*	*Trichophyton* sp.		hyphae
10	Skin	*Trichophyton indotineae*	*Trichophyton* sp.		hyphae
11	Nail	*Trichophyton interdigitale*	*Trichophyton* sp.		hyphae
12	Nail	*Trichophyton interdigitale*	*Trichophyton* sp.		neg
13	Hair	*Trichophyton mentagrophytes*	*Trichophyton mentagrophytes* complex/*Trichophyton interdigitale*		hyphae
14	Skin	*Trichophyton mentagrophytes* complex	*Trichophyton* sp.		neg
15	Nail	*Trichophyton rubrum*	*Trichophyton rubrum*		hyphae
16	Nail	*Trichophyton rubrum*	*Trichophyton rubrum*		hyphae
17	Nail	*Trichophyton rubrum*	*Trichophyton rubrum*		hyphae
18	Nail	*Trichophyton rubrum*	*Trichophyton rubrum*		hyphae
19	Nail	*Trichophyton rubrum*	*Trichophyton* sp.		hyphae
20	Skin	*Trichophyton rubrum*	*Trichophyton rubrum*		hyphae
21	Skin	*Trichophyton rubrum*	*Trichophyton rubrum*		hyphae
22	Skin	*Trichophyton rubrum*	*Trichophyton rubrum*		hyphae
23	Skin	*Trichophyton rubrum*	*Trichophyton rubrum*		hyphae
24	Skin	*Trichophyton rubrum*	*Trichophyton rubrum*		hyphae
25	Skin	*Trichophyton rubrum*	*Trichophyton rubrum*		hyphae
26	Skin	*Trichophyton rubrum*	*Trichophyton rubrum*		hyphae
27	Skin	*Trichophyton rubrum*	*Trichophyton rubrum*		hyphae
28	Skin	*Trichophyton rubrum*	*Trichophyton rubrum*		hyphae
29	Skin	*Trichophyton rubrum*	*Trichophyton rubrum*		hyphae
30	Skin	*Trichophyton rubrum*	*Trichophyton rubrum*		IQ
31	Skin	*Trichophyton rubrum*	*Trichophyton rubrum*		hyphae
32	Skin	*Trichophyton rubrum*	*Trichophyton rubrum*		hyphae
33	Skin	*Trichophyton rubrum*	*Trichophyton* sp.		hyphae

34	Skin	*Trichophyton rubrum*	*Trichophyton* sp.		hyphae
35	Skin	*Trichophyton rubrum*	*Trichophyton* sp.		hyphae
36	Skin	*Trichophyton rubrum*	*Trichophyton* sp.		IQ
37	Hair	*Trichophyton tonsurans*	*Trichophyton tonsurans*		neg
38	Hair	*Trichophyton violaceum*	*Trichophyton rubrum*	KO[1]	hyphae
39	Nail	*Trichophyton rubrum*, *Aspergillus* spp	*Trichophyton* sp.		hyphae
40	Skin	*Trichophyton rubrum*, *Candida parapsilosis*	*Trichophyton rubrum*, *Candida albicans*	*Trichophyton rubrum*	hyphae
41	Skin	*Trichophyton rubrum*, *Cladosporium* spp	*Trichophyton rubrum*		hyphae
42	Skin	*Trichophyton rubrum*, *Cladosporium* spp	*Trichophyton rubrum*		hyphae
43	Nail	*Trichophyton rubrum*, *Cryptococcus albidus*	*Trichophyton rubrum*		hyphae
44	Nail	*Trichophyton rubrum*, *Rhodotorula* spp	*Trichophyton rubrum*		hyphae
45	Skin	*Trichophyton rubrum*, *Rhodotorula mucilanoga*, *Cryptococcus diffluens*	Negative		hyphae
46	Skin	*Trichophyton mentagrophytes* complex	Negative		neg
47	Skin	*Trichophyton rubrum*	Negative		hyphae
48	Skin	*Trichophyton rubrum*	Negative		hyphae
49	Skin	*Trichophyton rubrum*	Negative		hyphae
50	Skin	*Trichophyton rubrum*	Negative		hyphae
51	Skin	*Trichophyton rubrum*	Negative		hyphae
52	Skin	*Trichophyton rubrum*, *Rhodotorula* spp	Negative		hyphae
53	Skin	*Trichophyton rubrum*, *Candida parapsilosis*	Negative		hyphae
54	Skin	*Trichophyton tonsurans*	Negative		hyphae
55	Nail	*Trichophyton rubrum*	Invalid PCR		hyphae
56	Skin	*Trichophyton mentagrophytes*	Invalid PCR		IQ
57	Skin	*Trichophyton rubrum*	Invalid PCR		hyphae
58	Nail	*Alternaria* spp	Negative		hyphae
59	Nail	*Fusarium* spp, *Cladosporium* spp, *Penicillium* spp	Negative		hyphae
60	Nail	*Scedosporium* spp, *Cladosporium* spp	Negative		hyphae
61	Skin	*Beauveria* spp	Negative		neg
62	Skin	Dematiaceous mold	Negative		neg
63	Nail	*Alternaria* spp	*Trichophyton rubrum*	KO[2]	hyphae
64	Nail	*Clonostachys* spp	*Trichophyton* sp.	*Trichophyton tonsurans*	neg
65	Skin	*Cladosporium* spp	Invalid PCR		neg
66	Nail	Sterile	Negative		neg
67	Nail	Sterile	Negative		neg

68	Nail	Sterile	Negative		neg
69	Skin	Sterile	Negative		neg
70	Skin	Sterile	Negative		neg
71	Skin	Sterile	Negative		neg
72	Nail	Sterile	Negative		neg
73	Nail	Sterile	Negative		hyphae
74	Nail	Sterile	Negative		hyphae
75	Skin	Sterile	Negative		hyphae
76	Skin	Sterile	Negative		hyphae
77	Nail	Sterile	*Trichophyton rubrum*	*Trichophyton rubrum*	hyphae
78	Nail	Sterile	*Trichophyton rubrum*	*Trichophyton rubrum*	hyphae
79	Nail	Sterile	*Trichophyton rubrum*	*Trichophyton rubrum*	hyphae
80	Nail	Sterile	*Trichophyton rubrum*	*Trichophyton rubrum*	hyphae
81	Nail	Sterile	*Trichophyton rubrum*	*Trichophyton rubrum*	hyphae
82	Skin	Sterile	*Trichophyton rubrum*	*Trichophyton rubrum*	hyphae
83	Skin	Sterile	*Trichophyton rubrum*	*Trichophyton rubrum*	neg
84	Nail	Sterile	*Trichophyton* sp.	KO[3]	neg
85	Nail	Sterile	Invalid PCR		neg

DME: direct microscopic examination; IQ: insufficient quantity; KO: unsuccessful Sanger sequencing; neg: negative; [1]MITS2a/2b sequence does not distinguish *T.violaceum* from *T.rubrum*; [2]Sequence mixture not analysable by Sanger sequencing; [3]Similarity percentage below 98%.
